# A novel approach to carotenoid accumulation in rice callus by mimicking the cauliflower *Orange* mutation via genome editing

**DOI:** 10.1186/s12284-019-0345-3

**Published:** 2019-11-12

**Authors:** Akira Endo, Hiroaki Saika, Miho Takemura, Norihiko Misawa, Seiichi Toki

**Affiliations:** 1 Plant Genome Engineering Research Unit, Institute of Agrobiological Sciences, National Agriculture and Food Research Organization, 3-1-3 Kannondai, Tsukuba, Ibaraki 305-8604 Japan; 2 Present Address of AE: Biotechnology Research Laboratories, KANEKA CORPORATION, 1-8, Miyamae-cho, Takasago-cho, Takasago, Hyogo 676-8688 Japan; 3 Research Institute for Bioresources and Biotechnology, Ishikawa Prefectural University, 1-308 Suematsu, Nonoichi-shi, Ishikawa 921-8836 Japan; 4 Kihara Institute for Biological Research, Yokohama City University, 641-12 Maioka-cho, Yokohama, 244-0813 Japan

**Keywords:** Cauliflower *Orange* mutant, Carotenoid, CRISPR/Cas9, Rice callus

## Abstract

**Background:**

β-carotene (provitamin A) is an important target for biofortification of crops as a potential solution to the problem of vitamin A deficiency that is prevalent in developing countries. A previous report showed that dominant expression of splicing variants in the *Orange* (*Or*) gene causes β-carotene accumulation in cauliflower curd. In this study, we focused on a putative orthologue of the cauliflower *or* gene in rice, *Osor*, and attempt to accumulate β-carotene in rice callus by modification of the *Osor* gene via genome editing using CRISPR/Cas9.

**Findings:**

CRISPR/Cas9 vectors for the *Osor* gene were constructed and transformed into rice calli. Some transformed calli showed orange color due to β-carotene hyper-accumulation. Molecular analyses suggest that orange-colored calli are due to an abundance of in-frame aberrant *Osor* transcripts, whereas out-of-frame mutations were not associated with orange color.

**Conclusions:**

We demonstrate that directed gene modification of the *Osor* gene via CRISPR/Cas9-mediated genome editing results in β-carotene fortification in rice calli. To date, golden rice, which accumulates β-carotene in rice endosperm, has been developed by conventional transgenic approaches. Our results suggest an alternative approach to enhancing β-carotene accumulation in crops.

## Findings

Genome editing techniques have been applied extensively in plant molecular breeding to generate crops with agronomically beneficial traits (Karkute et al. 2017). Biofortification of crops could help combat malnutrition by maximizing the production of various nutrients in staple crops. β-Carotene is an important target of biofortification in crops because it is a provitamin A, and an appropriate form to take up from vegetables such as carrot, sweetpotato, and spinach (Giuliano 2017). Since vitamin A is an essential nutrient for establishing vision and proper development in animals, including humans, β-carotene deficiency can cause pediatric blindness, which is still a major problem in developing countries (Klemm et al. 2010). Golden rice, which accumulates β-carotene in endosperm by expressing carotenoid biosynthesis genes under the control of an endosperm-specific promoter, was created to counter this problem (Paine et al. 2005). Here, we conceive a novel method to enhance accumulation of β-carotene in plants by use of the CRISPR/Cas9 system. The idea was inspired by observations of the cauliflower (*Brassica oleracea var. botrytis*) *Orange* (*Or*) mutant (Lu et al. 2006). Whereas wild-type (WT) cauliflower curd is normally white, the *Or* mutant produces orange-colored curd caused by high levels of β-carotene accumulation in non-photosynthetic tissues (Lu et al. 2006). The mutation leading to the carotenoid-accumulating phenotype is a retrotransposon insertion in the third exon of the *Or* gene that disrupts proper splicing of *Or* transcripts (Additional file 2: Figure S1). In the *Or* mutant, three types of aberrant transcripts are thought to function dominantly in abnormal accumulation of carotenoid by unknown mechanisms (Additional file 2: Figure S1) (Lu et al. 2006). From our knowledge of this phenomenon in the cauliflower *Or* mutant, we hypothesized that directed disruption of splicing of the *or* ortholog could reproduce the carotenoid-accumulating phenotype in other plant species. To test this hypothesis, we employed CRISPR/Cas9 to direct gene disruption of the *or* ortholog in rice callus via *Agrobacterium*-mediated transformation. The callus system is well-suited to evaluating the effects of genetic modification on carotenoid accumulation since callus is non-photosynthetic tissue in which carotenoid accumulation can be detected visually by callus color in various plant species such as sweetpotato, rice and *Arabidopsis* (Bai et al. 2014; Kim et al. 2013; Tzuri et al. 2015).

**Fig. 1:**
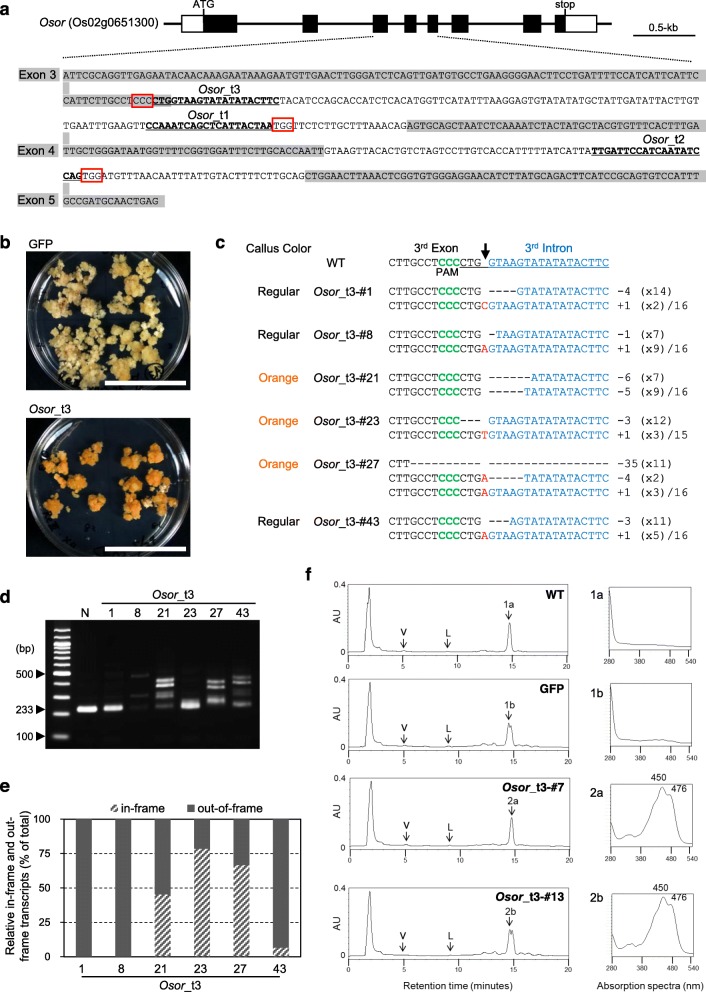
Reproduction of carotenoid-accumulating phenotype of cauliflower *Or* mutation in rice callus. **a** Target sequence in the *Osor* gene of each sgRNA for mimicking aberrant *or* transcripts expressed in the cauliflower *Or* mutation. Black and white boxes indicate exons and untranslated regions of the *Osor* gene, respectively. Sequences of exons are highlighted in gray; 20-nt target sequences are bold and underlined; 3-nt sequence indicating the protospacer adjacent motif (PAM) are boxed in red. **b** Photographs of hygromycin-resistant calli after 1-month of propagation. Upper and lower panels: calli from GFP and *Osor*_t3 lines, respectively. White bar = 5 cm. **c** Mutation patterns of *Osor*_t3 sgRNA loci. Sequences of 3rd exon and 3rd intron are presented in black and blue, respectively. Target DNA sequence of *Osor*_t3 sgRNA is shown in WT and underlined at the top of the aligned sequences. A black arrow indicates the expected digestion site by Cas9 with *Osor*_t3 sgRNA. The PAM sequence is indicated in green and bold. Deletions are indicated as dashes; insertions are in red. Indel size and number of clones are shown on the right (+, insertion; −, deletion; x, number of clones). **d** RT-PCR analysis of *OsOr* transcript in callus lines possessing mutations at *Osor*_t3 loci. Primers were designed to amplify the region from the 3rd exon to the 5th exon in the *Osor* gene. Expected lengths of PCR products amplified from cDNA or genomic DNA of *Osor* are 233 and 457 bp, respectively. N, non-transgenic plant. **e** Composition of in-frame or out-of-frame transcripts in each orange or regular-colored callus line, indicating the percentage of in-frame or out-of-frame transcripts in total sequenced clones shown in Additional file 2: Figure S10 and Additional file 3: Table S1. **f** Measurement of β-carotene content in orange callus. Left side: HPLC chromatograms of callus extracts. Right side: absorption spectra of the major peak in each chromatogram. V and L in each chromatogram indicate the expected retention times of violaxanthin and lutein, respectively. Materials and methods are listed in Additional files 1 and 3.

We first searched for the *or* ortholog gene in the rice genome database. Two genes (Os02g0651300 and Os02g0535000) whose products showed high similarity with the or protein were found (83% and 61% identities with or protein, respectively). RNA-seq data showed that expression of Os02g0651300 in calli was higher than that of Os02g0535000 (http://rice.plantbiology.msu.edu/index.shtml). Of these two predicted proteins, Os02g0651300 grouped together with or protein in a phylogenetic tree, whereas Os02g0535000 did not (Additional file 2: Figure S2). Therefore, we focused on Os02g0651300 as the most likely candidate of the rice ortholog of the *or* gene, and named it *Osor*. To produce a carotenoid-accumulating phenotype in rice calli by imitating mutations in the *or* gene, single guide RNAs (sgRNAs) were designed to mimic three types of aberrant transcripts found in the *Or* mutant (Additional file 2: Figure S1). The simultaneous use of *Osor*_t1 and t2 sgRNAs was expected to generate a deletion mutation emulating the *Or*_*LDel*_ transcript by removing the fourth exon (Fig. 1a and Additional file 2: Figure S1). Similarly, the *Osor*_t3 sgRNA should produce a mutation mimicking *Or*_*Ins*_ and *Or*_*Del*_ transcripts by disrupting the junction between the third exon and third intron (Figs. 1a and Additional file 2: Figure S1).

To examine the effect on carotenoid accumulation in rice callus of CRISPR/Cas9-induced targeted mutagenesis in the *Osor* gene, CRISPR/Cas9 constructs harboring Cas9, GFP and sgRNA expression cassettes (*Osor*_t1 and t2 sgRNAs or *Osor*_t3 sgRNA) (Additional file 2: Figure S3) were introduced into scutellum-derived calli of rice by *Agrobacterium*-mediated transformation. To distinguish independent targeted mutagenesis events, single pieces of callus from different callus clumps were transferred to fresh selection medium and propagated for genotyping analysis (Additional file 2: Figure S4). As shown in Fig. 1b and Additional file 2: Figure S5, orange callus was observed in hygromycin-resistant calli transformed with Cas9 and *Osor*_t3 sgRNA but not *Osor*_t1 and t2 sgRNAs. In some *Osor*_t1 and t2 sgRNA transformed lines, PCR for genotyping revealed two products of different size (Additional file 2: Figure S6); the smaller of these was caused by deletion between the regions targeted by *Osor*_t1 and t2 sgRNAs, including the fourth exon in the *Osor* genomic region (Additional file 2: Figure S7). RT-PCR analysis showed that the deletion resulted in truncated *OsOr* transcripts in lines #18 and #21 (Additional file 2: Figure S8). Although *Or*_*LDel*_-type *OsOr* transcripts were generated in some calli of *Osor*_t1 and t2 lines, no calli were orange in color. On the other hand, to detect mutations in orange-colored and regular calli of *Osor*_t3 lines, a heteroduplex mobility assay was performed (Additional file 2: Figure S9). Regardless of callus color, various mutations occurred around the junction between the third exon and third intron (Fig. 1c), and RT-PCR analysis showed that multiple *OsOr* transcripts were observed in most *Osor*_t3 lines, similar to the cauliflower *Or* mutant (Fig. 1d). Sequence analysis of *OsOr* transcripts in *Osor*_t3 lines revealed that most transcripts containing random partial sequences of the third intron between the third and fourth exon were caused by mutation-induced mis-splicing of *Osor* (Additional file 2: Figure S10, Additional file 3: Table S1). Interestingly, some of the mis-spliced transcripts are in-frame, and thus are expected to be translated into aberrant proteins where unknown polypeptides are inserted into WT or protein. We checked the correlation of callus color with such “aberrant in-frame transcripts” as described above. In orange-colored calli (*Osor*_t3-#21, #23 and #27), > 40% of *Osor* transcripts were in-frame aberrant transcripts including a small deletion of the third exon or insertion of the partial third intron between the third and fourth exon (Fig. 1e). In contrast, the majority of aberrant transcripts in regular-colored calli (*Osor*_t3-#1, #8 and #43) were out-of-frame (Fig. 1e). Therefore, loss-of-function mutations in *Osor* did not change callus color. Also, the difference between orange-colored and regular calli depended on the proportion of in-frame aberrant *OsOr* transcripts.

Since the cauliflower *Or* mutant accumulates high amounts of β-carotene in orange-colored curd (Lu et al. 2006), carotenoids in orange-colored, WT and control rice calli were extracted and analyzed by high performance liquid chromatography (HPLC). A major peak with a retention time similar to that of β-carotene was detected at 14.5–14.9 min in all genotypes (Fig. 1f). Absorption spectra of the major peak in each genotype indicated that β-carotene accumulated in orange calli from *Osor*_t3 lines but not in calli from the other genotypes (Fig. 1f). In calli from *Osor*_t3-#7 and *Osor*_t3-#13 lines, the levels of β-carotene were 2.73 ± 0.44 and 2.86 ± 1.41 μg/g fresh weight, respectively. In WT and control calli, the amount of β-carotene was below the detection limit. However, tiny amounts of violaxanthin and lutein were detected at the expected retention times in all calli tested (Fig. 1f). These results demonstrate that β-carotene accumulated prominently in orange-colored calli, as observed in curd of the cauliflower *Or* mutant.

In this study, we succeeded in reproducing the molecular and coloring phenotypes of the cauliflower *Or* mutant in rice calli by disrupting the junction between the third exon and third intron in the *Osor* gene using the CRISPR/Cas9 system. We designed sgRNAs to generate three types of aberrant *OsOr* transcripts: *Or*_*LDel*_, *Or*_*Ins*_ and *Or*_*Del*_. *Or*_*LDel*_ type *OsOr* transcripts did not cause accumulation of β-carotene in rice callus (Fig. 1b). As Or_LDel_ protein is not detected in the cauliflower *Or* mutant (Lu et al. 2006), the O_rLDel_ type OsOr protein might not contribute to accumulation of β-carotene in rice callus. Carotenoid accumulation in rice calli was correlated with the abundance of *Or*_*Ins*_ or *Or*_*Del*_-type in-frame *OsOr* transcripts (Fig. 1e). Although 39- and 21-bp footprints of the retrotransposon remain in cauliflower *Or*_*Ins*_ and *Or*_*Del*_ transcripts, in our study *Or*_*Ins*_ and *Or*_*Del*_ type *OsOr* transcripts have a small deletion of the third exon or partial insertion of the third intron. Therefore, the residual retrotransposon sequences are not necessary, and disruption of an unknown domain structure consisting of the third and fourth exons of *Osor* is sufficient for carotenoid accumulation in rice callus.

Rice endosperm, which does not produce carotenoids normally, has been shown to include geranylgeranyl diphosphate (GGPP) as a carotenoid precursor and to have the capability of expressing some endogenous carotenoid biosynthesis genes, e.g., lycopene β-cyclase gene (*LCYb*) (Bai et al. 2014; Paine et al. 2005). ‘Golden Rice 2’, which biosynthesizes β-carotene efficiently in the endosperm, was created by introducing the maize (*Zea mays*) phytoene synthase gene (*PSY*) and the soil-bacterial (*Pantoea ananatis*) phytoene desaturase gene (*crtI*), which mediate the biosynthesis of lycopene from GGPP, under the control of the endosperm-specific glutelin promoter (Paine et al. 2005). Previous studies have shown that not only PSY but also or proteins play a crucial role in effective accumulation of β-carotene. For example, it was revealed that the Arabidopsis *or* gene encodes a posttranscriptional regulator to interact with PSY, and an amino-terminal part of the sweetpotato or protein acts as a molecular chaperone possessing holdase activity to inhibit aggregation of PSY (Park et al. 2016; Zhou et al. 2015). Overexpression of *Ator* with carotenoid biosynthesis genes *PSY* and *crtI* enhanced the accumulation of carotenoids in rice callus and maize seeds (Bai et al. 2014; Berman et al. 2017). Overexpression of a mutated Or protein led to higher levels of carotenoid accumulation than with WT or protein in potato tuber and sweetpotato callus (Kim et al. 2013; Lu et al. 2006). Thus, modification of an individual gene and/or its expression levels in either endogenous carotenoid biosynthesis genes or the *or* gene via genome editing might be useful for carotenoid accumulation in rice endosperm. It may also be feasible to increase β-carotene levels in rice seeds of *Osor*-edited and null-segregant plants, although further analyses will be needed. Finally, we demonstrated that genome editing of *Osor* allowed rice callus to increase levels of β-carotene from undetectable to easily measurable. As presented here, genome editing of *or* orthologs could be applied to various crops to boost the accumulation of β-carotene in the edible parts of crops in which carotenoid biosynthesis occurs without introducing any carotenoid biosynthetic genes as transgenes.

## Supplementary information

**Additional file 1.** Materials and Methods.

**Additional file 2: Figure S1.** Schematic view of cauliflower *Or* mutation causing carotenoid accumulation in curd. **Figure S2.** Phylogenetic tree of or orthologs from various plant species. **Figure S3.** T-DNA construction and sgRNA expression cassette introduction into gateway cassette in T-DNA. **Figure S4.** Experimental scheme from transformation to genotyping of *Osor* targeted loci. **Figure S5.** Photographs of hygromycin resistant calli from GFP, *Osor*_t1 and t2, and *Osor*_t3 lines. **Figure S6.** Detection of deletion at *Osor*_t1 and t2 loci. **Figure S7.** Mutation patterns at *Osor*_t1 and t2 loci. **Figure S8.** RT-PCR analysis of *OsOr* transcript in calli of *Osor*_t1 and t2 lines. **Figure S9**. Heteroduplex mobility assay to detect mutation in *Osor*_t3 locus. **Figure S10.** Mutation patterns of *OsOr* transcripts from each callus line harboring *Osor*_t3.

**Additional file 3: Table S1.** Sequences of *OsOr* transcripts from each callus line harboring *Osor*_t3. **Table S2.** List of or orthologs from various plant species. **Table S3.** Primers used in this study.

## Data Availability

The datasets supporting the conclusions of this article are included within the article and its additional files.

## Abbreviations

HPLC: High performance liquid chromatography
or: orange
PAM: Protospacer adjacent motif
PSY: Phytoene synthase
sgRNA: single guide RNA
WT: Wild-type
