# Supplementary material for: A novel approach to carotenoid accumulation in rice callus by mimicking the cauliflower Orange mutation via genome editing
Source: Rice (N Y). 2019 Nov 12;12:81. doi: 10.1186/s12284-019-0345-3 (PMC6851270; doi:10.1186/s12284-019-0345-3)
Supplement: Supplementary file 1 — Additional file 1. Materials and Methods. [file 12284_2019_345_MOESM1_ESM.docx]

**Supplemental Materials and Methods**

**Phylogenetic tree analysis**

Amino acid sequences of cauliflower or orthologues were collected from the Phytozome database (<https://phytozome.jgi.doe.gov/pz/portal.html>). A phylogenetic tree was constructed using MEGA7 (Kumar et al. 2016). Alignment of 74 full-length amino acid sequences from 56 plant species resulted in a maximum-likelihood tree with default settings. Initial tree(s) for the heuristic search were obtained automatically by applying the Neighbor-Joining and BioNJ algorithms to a matrix of pairwise distances estimated using a Jones-Thornton-Taylor (JTT) model, and then selecting the topology with superior log likelihood value. Sequence IDs of all sequences are listed in Table S2.

**Vector construction and cloning of guide RNA**

Two DNA fragments of PcUbi pro::SpCas9 and 2x35S pro::HPT were amplified by PCR from pDe-Cas9 and pMDC32, respectively (Curtis and Grossniklaus 2003; Fauser et al. 2014). PcUbi represents the *ubiquitin 4-2* promoter region from *Petroselinum crispum* (Kawalleck et al. 1993)*.* PCR products were cloned into the T-DNA region of the binary vector pPZP200 (Hajdukiewicz et al. 1994). The gateway attR cassette was inserted between PcUbi pro::SpCas9 and 2x35S pro::HPT fragments.

DNA oligos (Table S3) to express sgRNA targeting for *Osor* gene were cloned into the *Bbs*I site under control of the *U6-2* promoter from rice in entry vectors possessing attL gateway recombination sequences. The U6 pro::sgRNA cassette was moved to the binary vector by LR reaction as described in manufacturer’s guide (Fig. S3).

**Rice transformation**

The resulting binary vectors were introduced into *Agrobacterium* strain EHA105 (Hood et al. 1993). *Agrobacteria* harboring the binary vector were used for transformation of calli derived from scutellum in mature rice seeds (*Oryza sativa* L. cv Nipponbare). Details of the transformation procedure were described previously (Toki et al. 2006).

**DNA extraction and genotyping**

DNA was isolated from rice calli using an Agencourt chloropure kit (Beckman Coulter). To detect mutations, PCR was performed with specific primer sets (Table S3). PCR products were analyzed by agarose gel electrophoresis to detect deletion mutations. The heteroduplex mobility assay was performed using a microchip electrophoresis system, MultiNA (Shimadzu).

**RNA extraction and reverse transcription (RT)-PCR**

RNA was extracted using an RNeasy kit (QIAGEN). RNA samples were subjected to DNase I treatment (TaKaRa). Single-strand DNA was synthesized with ReverTra Ace (TOYOBO). RT-PCR was executed using a specific primer set for *Osor* (Table S3). To estimate the levels of in-frame and out-of-frame transcripts, cDNA was cloned into pCR Blunt II-TOPO vector (Invitrogen) and subjected to sequence analysis using an ABI3500 sequencer (Applied Biosystems).

**Carotenoid measurement**

Mature seeds from T1 plants regenerated from orange-colored or control calli were used to induce scutellum-derived calli on N6D medium. Calli were separated from embryos and endosperm, and 0.3 g secondary calli was collected from each line and subjected to HPLC analysis to determine carotenoid content. The calli were ground in liquid N2 and suspended in 100 μL of methanol. 50 μL of 50 mM Tris-HCl (pH 7.5), 150 μL of 1M NaCl, and 200 μL of chloroform were added to the solution, mixed for 5 min, and centrifuged. The lower phase was collected and dried by centrifugal evaporation. The pellet was suspended with ethyl acetate and analysed using HPLC-PDA. HPLC analysis was performed using a Waters Alliance 2695-2996 system with a photo diode array detector as described previously (Takemura et al. 2015). A TSKgel ODS-80s column (4.6 × 150 nm, 5 μg; Tosoh) was used. Samples were eluted at a flow rate of 1.0 ml/min at 25°C with two solvents – solvent A (water-methanol, 5:95, v/v) and solvent B (tetrahydrofuran-methanol, 30:70, v/v). Solvent A was perfused for 5 min, followed by a linear gradient from solvent A to solvent B for 5 min, and then solvent B alone for 8 min. Carotenoids were identified by comparing retention times and absorbing spectra with authentic standards. All chromatograms are shown with each peak at its λ_max_ (Maxplot).

**References**

Curtis MD, Grossniklaus U (2003) A gateway cloning vector set for high-throughput functional analysis of genes in planta. Plant Physiol. 133: 462-469.

Fauser F, Schiml S, Puchta H (2014) Both CRISPR/Cas-based nucleases and nickases can be used efficiently for genome engineering in *Arabidopsis thaliana*. Plant J. 79: 348-359.

Hajdukiewicz P, Svab Z, Maliga P (1994) The small, versatile pPZP family of *Agrobacterium* binary vectors for Plant transformation. Plant Mol. Biol. 25: 989-994.

Hood EE, Gelvin SB, Melchers LS, Hoekema A (1993) New *Agrobacterium* helper plasmids for gene transfer to plants. Transgenic Res. 2: 208-218.

Kawalleck P, Somssich IE, Feldbrugge M, Hahlbrock K, Weisshaar B (1993) Polyubiquitin gene expression and structural properties of the *ubi*4-2 gene in *Petroselinum crispum*. Plant Mol. Biol. 21: 673-684.

Kumar S, Stecher G, Tamura K (2016) MEGA7: Molecular Evolutionary Genetics Analysis Version 7.0 for Bigger Datasets. Mol. Biol. Evol. 33: 1870-1874.

Takemura M, Maoka T, Misawa N (2015) Biosynthetic routes of hydroxylated carotenoids (xanthophylls) in *Marchantia polymorpha*, and production of novel and rare xanthophylls through pathway engineering in *Escherichia coli*. Planta 241: 699-710.

Toki S, Hara N, Ono K, Onodera H, Tagiri A, Oka S, Tanaka H (2006) Early infection of scutellum tissue with *Agrobacterium* allows high-speed transformation of rice. Plant J. 47: 969-976.
