# Supplementary material for: A novel approach to carotenoid accumulation in rice callus by mimicking the cauliflower Orange mutation via genome editing
Source: Rice (N Y). 2019 Nov 12;12:81. doi: 10.1186/s12284-019-0345-3 (PMC6851270; doi:10.1186/s12284-019-0345-3)
Supplement: Supplementary file 2 — Additional file 2: Figure S1. Schematic view of cauliflower Or mutation causing carotenoid accumulation in curd. Figure S2. Phylogenetic tree of or orthologs from various plant species. Figure S3. T-DNA construction and sgRNA expression cassette introduction into gateway cassette in T-DNA. Figure S4. Experimental scheme from transformation to genotyping of Osor targeted loci. Figure S5. Photographs of hygromycin resistant calli from GFP, Osor_t1 and t2, and Osor_t3 lines. Figure S6. Detection of deletion at Osor_t1 and t2 loci. Figure S7. Mutation patterns at Osor_t1 and t2 loci. Figure S8. RT-PCR analysis of OsOr transcript in calli of Osor_t1 and t2 lines. Figure S9. Heteroduplex mobility assay to detect mutation in Osor_t3 locus. Figure S10. Mutation patterns of OsOr transcripts from each callus line harboring Osor_t3. [file 12284_2019_345_MOESM2_ESM.pdf]

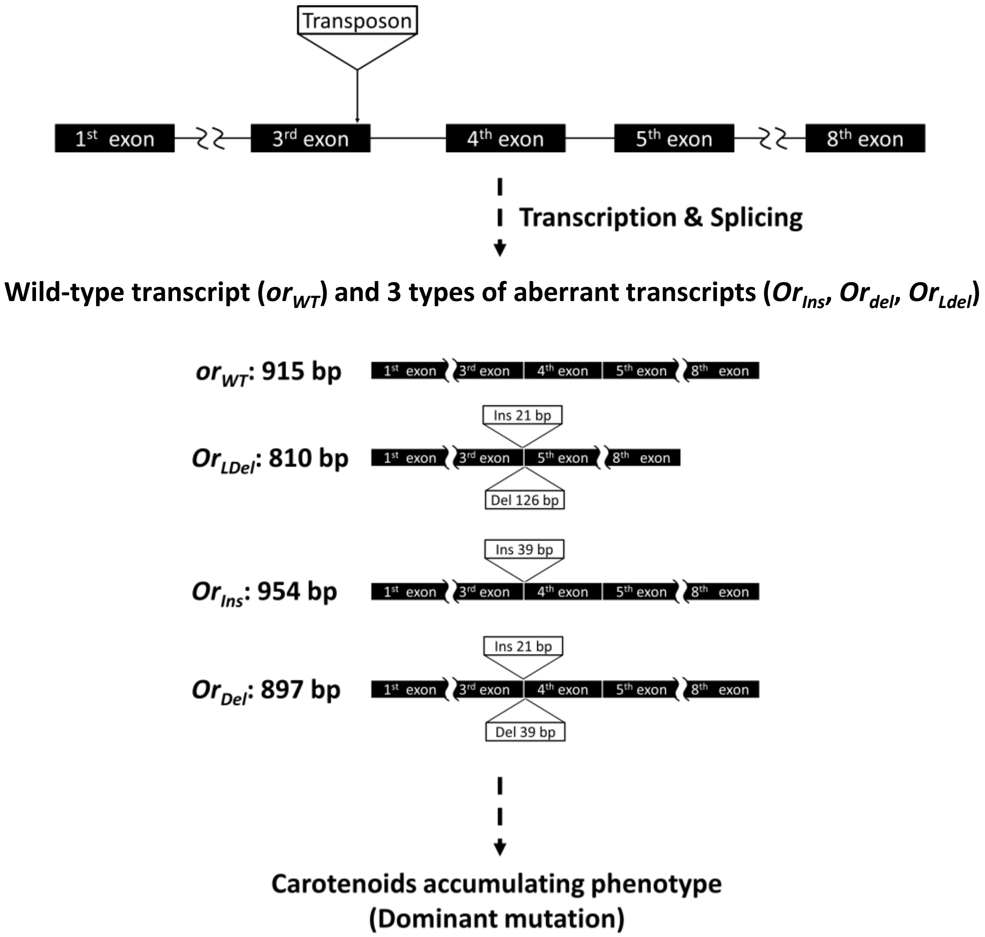

Figure S1. Schematic view of cauliflower *Or* mutation causing carotenoid accumulation in curd.

orange (*or*) gene structure is shown by black boxes (exons) and solid lines (introns). “Transposon” represents the retrotransposon inserted into the 3rd exon in *or* gene. Transposon insertion induces mis-splicing of the *or* transcript. Three types of aberrant *Or* transcripts observed in cauliflower *Or* mutant. Or_{LDel} contains a 21-bp footprint from the retrotransposon and a large deletion of 126-bp causing loss of the fourth exon. Or_{Ins} have 39-bp of insertion from retrotransposon. Or_{Del} has 21-bp of remaining sequence from the retrotransposon and a 39-bp deletion. Part of this figure was prepared based on a report by Lu *et al*. (2006).

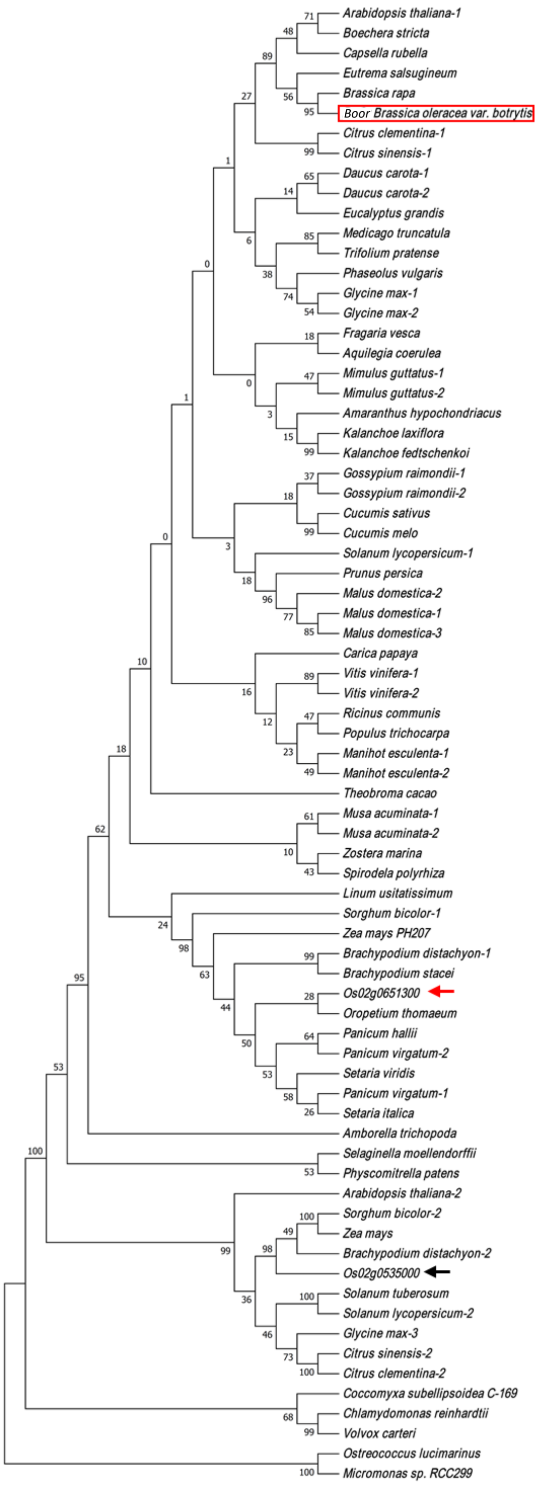

Figure S2. Phylogenetic tree of or orthologs from various plant species.

Seventy-four amino acid sequences from 56 species were used to construct the phylogenetic tree using the Maximum Likelihood method. The percentage of trees in which the associated taxa clustered together is shown next to the branches. Phylogeny test was performed using the bootstrap method with 500 replications. Cauliflower or protein is marked by a red box as Boor. Os02g0651300 and Os02g0535000 are indicated by red and black arrows, respectively.

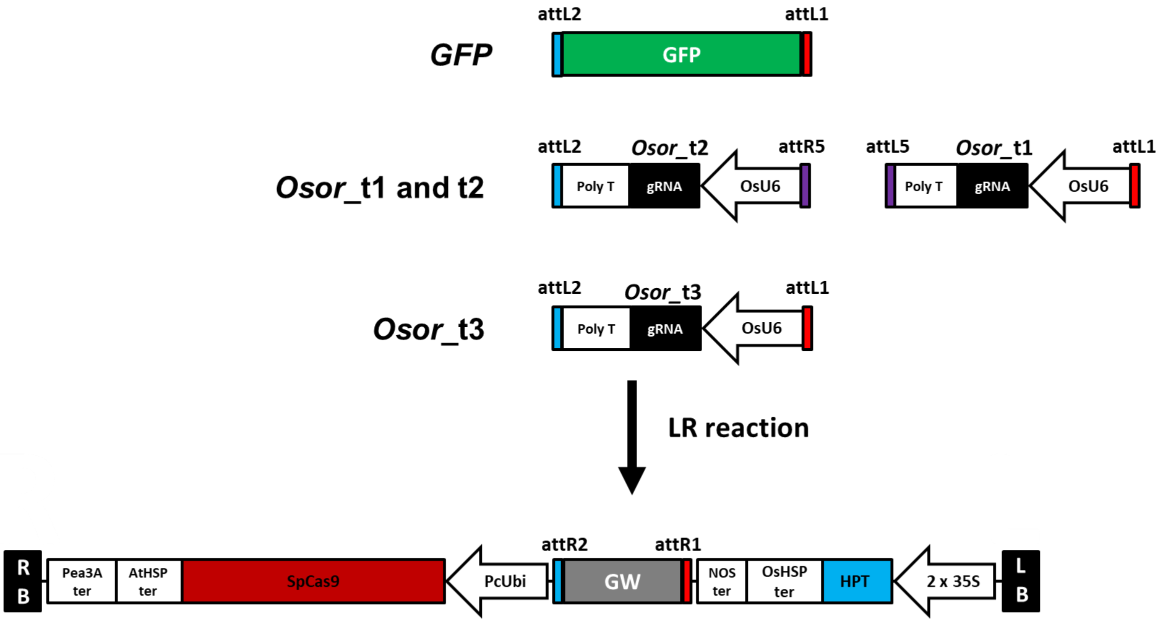

Figure S3. T-DNA construction and sgRNA expression cassette introduction into gateway cassette in T-DNA.

GFP: GFP coding sequence was inserted into the entry vector to create a binary vector as a negative control. *Osor*_t1 & t2: two sgRNA sequences targeting the *Osor*_t1 and t2 loci were independently cloned under control of the rice *U6-2* promoter of the entry vector. *Osor*_t3: a sgRNA targeting for the *Osor*_t3 locus was cloned under the control of the rice *U6-2* promoter of the entry vector. The resulting gateway cassettes in each entry vector were transferred to the binary vector using LR clonase. OsU6; rice *U6-2* promoter, PolyT; polyadenylation signal sequences, 2x35S: double 35S promoter, HPT: *hygromycin phosphotransferase*, OsHSP ter: terminator of *Oryza sativa heat shock protein 17-3*, NOS ter: terminator of *nopaline synthase*, GW: gateway cassette containing *ccdB* gene and *chloramphenicol acetyltransferase*, PcUbi: *Petroselinum crispum ubiquitin* promoter, SpCas9; *Streptococcus pyogenes* Cas9, AtHSP ter; the terminator region of *Arabidopsis thaliana HEAT SHOCK PROTEIN 18.2* gene, Pea3A ter; the terminator region of *Pisum sativum rbcS 3A* gene.

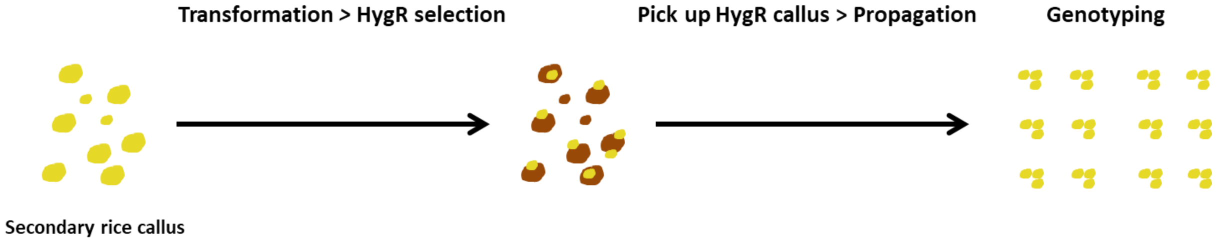

Figure S4. Experimental scheme from transformation to genotyping of *Osor* targeted loci.

Primary callus derived from mature seeds was cultivated on callus induction medium. Secondary callus derived from primary callus was infected with *Agrobacterium* harboring the vector shown in Fig. S3 and successfully transformed cells were selected with hygromycin. Hygromycin-resistant calli were clonally propagated and their genotype was checked.

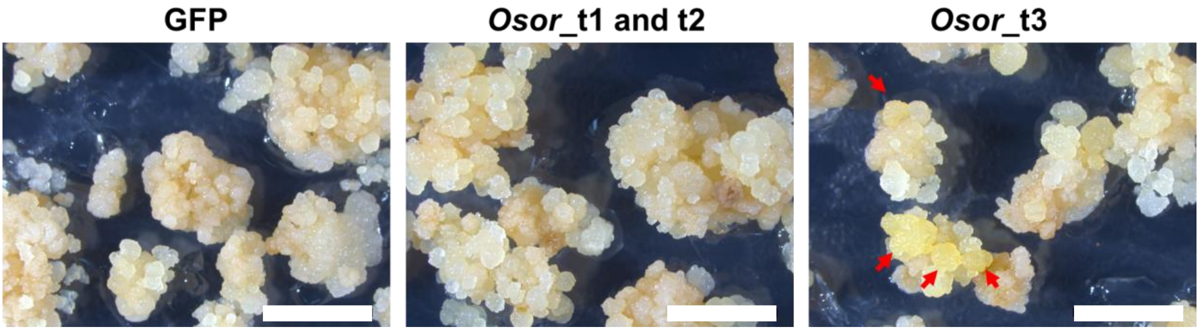

Figure S5. Photographs of hygromycin resistant calli from *GFP*, *Osor*_t1 and t2, and *Osor*_t3 lines.

Photographs of emerging hygromycin resistant calli. The genotype of each callus is represented above the photographs. A red arrowhead indicates orange-colored callus. White bar = 5 mm.

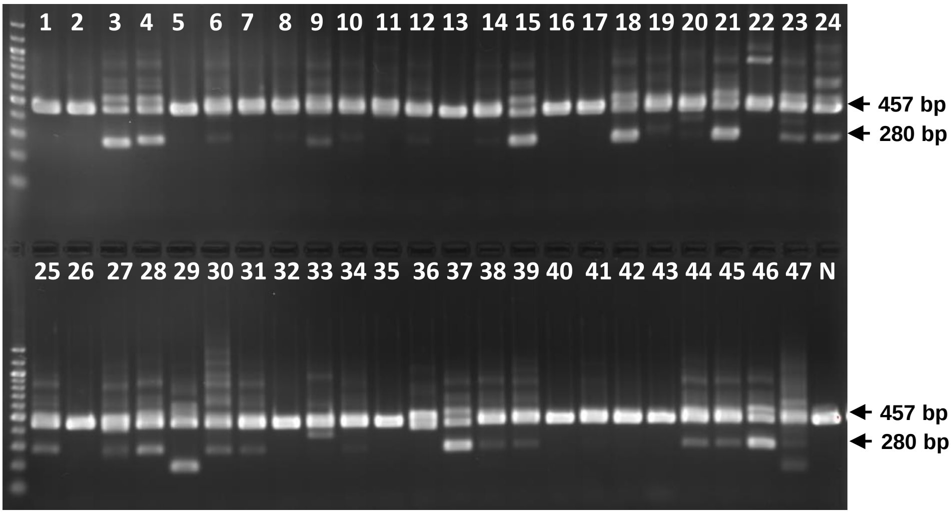

Figure S6. Detection of deletion at *Osor*_t1 and t2 loci.

#1-47 indicate independent hygromycin resistant calli harboring *Osor*_t1 and t2 sgRNAs. N, non-transgenic rice calli. The PCR product amplified from genomic DNA is 457 bp. *Osor*_t1 and t2 sgRNAs caused deletion of approximately 170 bp in the *Osor* gene. A 280-bp PCR product indicates that the expected deletion occurred in *Osor*_t1 and t2 loci.

```
                 Osor_t1                                    Osor_t2
                    ↓                                          ↓
WT CCAAATCAGCTCATTAC TAATGG-----//-----TTGATTCCATCAATATC CAGTGG

Osor_t1&t2-#3
   CCAAATCAGCTCATTACCTAATGG-----//-----TTGATTCCATCAATATCTCAGTGG   +2(x7)
   CCAAATCAG-------- -----------//---------------------- ---TGG -181(x9)

Osor_t1&t2-#4
   CCAAATCAGCTCATTAC TAATGG-----//-----TTGATTCCATCAATATC -AGTGG   -1(x2)
   CCAAATCAGCTCATTAC TAATGG-----//-----TTGATTCCATCAATATCTCAGTGG   +1(x2)
   CCAAATCAGCTCATTACTTAATGG-----//-----TTGATTCCATCAATATC -AGTGG    0(x1)
   CCAAATCAGCTCATTAC -----------//---------------------- CAGTGG -170(x9)

Osor_t1&t2-#15
   CCAAAT-------------AATGG-----//-----TTGATTCCATCAATATCCCAGTGG  -17(x9)
   CCAAATCAGCTCAT-A- -----------//---------------------- CAGTGG -172(x7)

Osor_t1&t2-#18
   CCAAATCAGCTCATTACATAATGG-----//-----TTGATTCCATCAAT--- CAGTGG   -2(x1)
   CCAAATCAGCTCATTACTTAATGG-----//-----TTGATTCCATCAAT--- CAGTGG   -2(x1)
   CCAAATCAGCTCATTACCTAATGG-----//-----TTGATTCCATCAAT--- CAGTGG   -2(x1)
   CCAAATCAGCTCATTAC TAATGG-----//-----TTGATTCCATCAAT--- CAGTGG   -3(x2)
   ----------------- -----------//-----TTGATTCCATCAAT--- CAGTGG  -48(x1)
   ----------------- -----------//-----TTGATTCCATCAAT--- CAGTGG  -49(x1)
   CCAAATCAGCTCATTAC -----------//---------------------- CAGTGG -170(x9)

Osor_t1&t2-#21
   CCAAATCAGCTCATTAC TAATGG-----//-----TTGATTCCATCAATATCTCAGTGG   +1(x4)
   CCAAATCAGCTCATTACCTAATGG-----//-----TTGATTCCATCAATATCTCAGTGG   +2(x1)
   CCAAATCAGCTCATTACA-----------//---------------------- CAGTGG -169(x11)
```

Figure S7. Mutation patterns at *Osor*_t1 and t2 loci.

The targeted regions of *Osor*_t1 and t2 are shown in wild-type (WT) at the top (underlined). Green characters indicate protospacer adjacent motif (PAM). Arrows indicate expected Cas9 digestion site. Red dashes represent deletion; red characters represent insertion. Line numbers of each callus were indicated at the left side of aligned sequences. The length of indel and the number of clones are represented at the right side of each sequence.

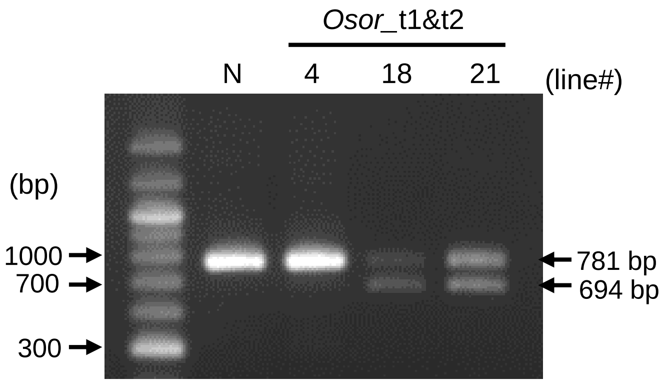

Figure S8. RT-PCR analysis of *OsOr* transcript in calli of *Osor*_t1 and t2 lines.

Primers were designed to amplify the region from 1st exon to 8th exon in *Osor* gene in *Osor*_t1&t2-#4, 18, and 21. The expected lengths of PCR products amplified from cDNA or genome DNA of *Osor* were 781 or 2683 bp, respectively. Expected deletion mutation of *Osor* results in a 694-bp PCR product. N, non-transgenic plant.

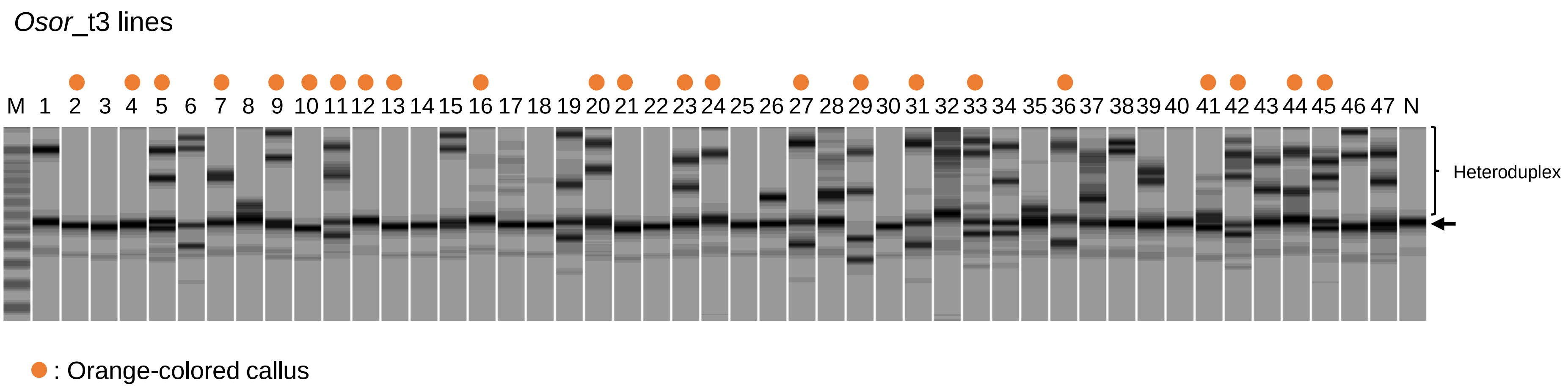

Figure S9. Heteroduplex mobility assay to detect mutation in *Osor*_t3 locus.

#1-47 indicate independent hygromycin resistant calli harboring *Osor*_t3 sgRNA. Orange dots indicate calli showing orange color. An arrow indicates the expected size of PCR product amplified from genomic DNA by specific primer set (*Osor* 3rd exon F and *Osor* 5th exon R). N, non-transgenic rice calli

Callus Color		3rd Exon		4th Exon	
	WT	ATTCCATTCTTGCCTCCCCTG		AGTGCAGCTAATCTCAAAATC	
		I P F L P P L		S A A N L K I	
**Regular**	*Osor*_t3-#1	ATTCCATTCTTGCCTCCCCTG	A	AGTGCAGCTAATCTCAAAATC	+1 (x5)
		ATTCCATTCTTGCCTCCCCT-		AGTGCAGCTAATCTCAAAATC	-1 (x9)/14
**Regular**	*Osor*_t3-#8	ATTCCATTCTTGCCTCCCCTG	A	AGTGCAGCTAATCTCAAAATC	+1 (x4)
		ATTCCATTCTTGCCTCCCCTG	...(+113bp)...	---------------------	*[1] (x5)
		ATTCCATTCTTGCCTCCCCTG	...(+137bp)...	AGTGCAGCTAATCTCAAAATC	*[2] (x1)
		ATTCCATTCTTGCCTCCCCTG	...(+138bp)...	AGTGCAGCTAATCTCAAAATC	*[2] (x2)
		ATTCCATTCTTGCCTCCCCTG	...(+125bp)...	AGTGCAGCTAATCTCAAAATC	*[2] (x1)/13
**Orange**	*Osor*_t3-#21	ATTCCATTCTTGCCTCCCCT-		AGTGCAGCTAATCTCAAAATC	-1 (x1)
		ATTCCATTCTTGCCTCCCCTG	...(+34bp)....	AGTGCAGCTAATCTCAAAATC	+34 (x1)
		ATTCCATTCTTGCCTCCCCTG	...(+47bp)....	AGTGCAGCTAATCTCAAAATC	+47 (x2)
		ATTCCATTCTTGCCTCCCCTG	...(+48bp)....	AGTGCAGCTAATCTCAAAATC	+48 (x3)
		ATTCCATTCTTGCCTCCCCTG	...(+51bp)....	AGTGCAGCTAATCTCAAAATC	+51 (x2)
		ATTCCATTCTTGCCTCCCCTG	...(+135bp)...	AGTGCAGCTAATCTCAAAATC	+135 (x2)/11
**Orange**	*Osor*_t3-#23	ATTCCATTCTTGCCTCCCCTG	T	AGTGCAGCTAATCTCAAAATC	+1 (x3)
		ATTCCATTCTTGCCTCCC---		AGTGCAGCTAATCTCAAAATC	-3 (x11)/14
**Orange**	*Osor*_t3-#27	ATTCCATTCTTGCCTCCCCTG	A	AGTGCAGCTAATCTCAAAATC	+1 (x2)
		ATTCCATTCTT----------	...(+28bp)....	AGTGCAGCTAATCTCAAAATC	-10/+28 (x8)
		ATTCCATTCTT----------	...(+112bp)...	AGTGCAGCTAATCTCAAAATC	-10/+112 (x2)/12
**Regular**	*Osor*_t3-#43	ATTCCATTCTTGCCTCCCCTG	A	AGTGCAGCTAATCTCAAAATC	+1 (x6)
		ATTCCATTCTTGCCTCCCCTG	A	-------CTAATCTCAAAATC	+1/-7 (x1)
		ATTCCATTCTTGCCTCCCCTG	...(+37bp)....	AGTGCAGCTAATCTCAAAATC	+37 (x1)
		ATTCCATTCTTGCCTCCCCTG	...(+50bp)....	AGTGCAGCTAATCTCAAAATC	+50 (x5)
		ATTCCATTCTTGCCTCCCCTG	...(+134bp)...	AGTGCAGCTAATCTCAAAATC	+134 (x2)/15

Figure S10. Mutation patterns of *Osor* transcripts from each callus line harboring *Osor*_t3.

The junction sequences between the 3rd and 4th exon of *Osor* are shown and the *Osor* transcript without mutations are presented with WT at the top. Amino acids corresponding to each codon are showed under the WT sequence. Deletions are indicated as red dashes, insertions are in red. The indel size and the number of clones are shown on the right side (+, insertion; –, deletion; x, number of clones). In-frame aberrant transcripts are highlighted with yellow color. Sequences of *OsOr* transcripts here are shown in Table S1. *[1]; 113 and 38 bp fragments from 3rd and 4th introns were inserted in the transcripts, and there were no sequences of 4th exon due to exon skipping, *[2]; Almost completely intact fragments of 3rd and 4th intron remain in the transcripts.
