# Supplementary material for: A novel approach to carotenoid accumulation in rice callus by mimicking the cauliflower Orange mutation via genome editing
Source: Rice (N Y). 2019 Nov 12;12:81. doi: 10.1186/s12284-019-0345-3 (PMC6851270; doi:10.1186/s12284-019-0345-3)
Supplement: Supplementary file 3 — Additional file 3: Table S1. Sequences of OsOr transcripts from each callus line harboring Osor_t3. Table S2. List of or orthologs from various plant species. Table S3. Primers used in this study. [file 12284_2019_345_MOESM3_ESM.pdf]

Table S1. Sequences of *OsOr* transcripts from each callus line harboring *Osor*_t3.

Callus line	Transcript sequences (Fig. S10)	Expected amino acid sequences
WT	ATTCCATTCTTGCCTCCCCTGAGTGCAGCTAATCTCAAAATC	IPFLPPLSAANLKI
*Osor*_t3-#1	ATTCCATTCTTGCCTCCCCTGAAGTGCAGCTAATCTCAAAATC	IPFLPPLKCS*
	ATTCCATTCTTGCCTCCCCT-AGTGCAGCTAATCTCAAAATC	IPFLPPLVQLISKSTMLRVSL*
*Osor*_t3-#8	ATTCCATTCTTGCCTCCCCTGAAGTGCAGCTAATCTCAAAATC	IPFLPPLKCS*
	ATTCCATTCTTGCCTCCCCTGGTAAGTATATATACTTCTACATCCAGCACCATCTCACATGGTTCATATTTAAGGAGTGTATATATGCTATTGATATTACTTGTTGAATTTGAAGTTCCAAATCAGCTCATTACCAGTGGATGTTTAACAATTTATTGTACTTTTCTTGCAGCTGGAACTTAA (green letters indicate the sequences of 5th exon)	IPFLPPLVSIYTSTSSTISHGSYLRSVYMLLILLVEFEVPNQLITSGCLTIYCTFLAAGT*
	ATTCCATTCTTGCCTCCCCTGGTAAGTATATATACTTCTACATCCAGCACCATCTCACATGGTTCATATTTAAGGAGTGTATATATGCTATTGATATTACTTGTTGAATTTGAAGTTCCAAATCAGCTCATTACTAATGGTTCTCTTGCTTTAAACAGAGTGCAGCTAATCTCAAAATC	IPFLPPLVSIYTSTSSTISHGSYLRSVYMLLILLVEFEVPNQLITSGCLTIYCTFLAAGT*
	ATTCCATTCTTGCCTCCCCTGGTAAGTATATATACTTCTACATCCAGCACCATCTCACATGGTTCATATTTAAGGAGTGTATATATGCTATTGATATTACTTGTTGAATTTGAAGTTCCAAATCAGCTCATTACCTAATGGTTCTCTTGCTTTAAACAGAGTGCAGCTAATCTCAAAATC	IPFLPPLVSIYTSTSSTISHGSYLRSVYMLLILLVEFEVPNQLIT*
	ATTCCATTCTTGCCTCCCCTGGTAAGTATATATACTTCTACATCCAGCACCATCTCACATGGTTCATATTTAAGGAGTGTATATATGCTATTGATATTACTTGTTGAATTTGAAGTTCCAAATAATGGTTCTCTTGCTTTAAACAGAGTGCAGCTAATCTCAAAATC	IPFLPPLVSIYTSTSSTISHGSYLRSVYMLLILLVEFEVPNNGSLALNRVQLISKSTMLRVSL*
*Osor*_t3-#21	ATTCCATTCTTGCCTCCCCT-AGTGCAGCTAATCTCAAAATC	IPFLPPLVQLISKSTMLRVSL*
	ATTCCATTCTTGCCTCCCCTGATATATACTTCTACATCCAGCACCATCTCACATGAGTGCAGCTAATCTCAAAATC	IPFLPPLIYTSTSSTISHECS*
	ATTCCATTCTTGCCTCCCCTGATATATACTTCTACATCCAGCACCATCTCACATGGTTCATATTTAAGAGTGCAGCTAATCTCAAAATC	IPFLPPLIYTSTSSTISHGSYLRVQLISKSTMLRVSL*
	ATTCCATTCTTGCCTCCCCTGTATATATACTTCTACATCCAGCACCATCTCACATGGTTCATATTTAAGAGTGCAGCTAATCTCAAAATC	IPFLPPLYIYFYIQHHLTWFIFKSAANLKI
	ATTCCATTCTTGCCTCCCCTGAAGTATATATACTTCTACATCCAGCACCATCTCACATGGTTCATATTTAAGAGTGCAGCTAATCTCAAAATC	IPFLPPLKYIYFYIQHHLTWFIFKSAANLKI
	ATTCCATTCTTGCCTCCCCTGAAGTATATATACTTCTACATCCAGCACCATCTCACATGGTTCATATTTAAGGAGTGTATATATGCTATTGATATTACTTGTTGAATTTGAAGTTCCAAATCAGCTCATTACTAATGGTTCTCTTGCTTTAAACAGAGTGCAGCTAATCTCAAAATC	IPFLPPLKYIYFYIQHHLTWFIFKECIYAIDITC*
*Osor*_t3-#23	ATTCCATTCTTGCCTCCCCTGTAGTGCAGCTAATCTCAAAATC	IPFLPPL*
	ATTCCATTCTTGCCTCCC---AGTGCAGCTAATCTCAAAATC	IPFLPP-SAANLKI
*Osor*_t3-#27	ATTCCATTCTTGCCTCCCCTGAAGTGCAGCTAATCTCAAAATC	IPFLPPLKCS*
	ATTCCATTCTT----------GCACCATCTCACATGGTTCATATTTAAGAGTGCAGCTAATCTCAAAATC	IPFLHHLTWFIFKSAANLKI
	ATTCCATTCTT----------GCACCATCTCACATGGTTCATATTTAAGGAGTGTATATATGCTATTGATATTACTTGTTGAATTTGAAGTTCCAAATCAGCTCATTACTAATGGTTCTCTTGCTTTAAACAGAGTGCAGCTAATCTCAAAATC	IPFLHHLTWFIFKECIYAIDITC*
*Osor*_t3-#43	ATTCCATTCTTGCCTCCCCTGAAGTGCAGCTAATCTCAAAATC	IPFLPPLKCS*
	ATTCCATTCTTGCCTCCCCTGA-------CTAATCTCAAAATC	IPFLPPL--TNLKI
	ATTCCATTCTTGCCTCCCCTGAGTATATATACTTCTACATCCAGCACCATCTCACATGAGTGCAGCTAATCTCAAAATC	IPFLPPLSIYTSTSSTISHECS*
	ATTCCATTCTTGCCTCCCCTGAGTATATATACTTCTACATCCAGCACCATCTCACATGGTTCATATTTAAGAGTGCAGCTAATCTCAAAATC	IPFLPPLSIYTSTSSTISHGSYLRVQLISKSTMLRVSL*
	ATTCCATTCTTGCCTCCCCTGAGTATATATACTTCTACATCCAGCACCATCTCACATGGTTCATATTTAAGGAGTGTATATATGCTATTGATATTACTTGTTGAATTTGAAGTTCCAAATCAGCTCATTACTAATGGTTCTCTTGCTTTAAACAGAGTGCAGCTAATCTCAAAATC	IPFLPPLSIYTSTSSTISHGSYLRSVYMLLILLVEFEVPNQLITNGSLALNRVQLISKSTMLRVSL*

Table S2. List of or orthologs from various plant species

Species	Sequence ID	Species	Sequence ID
Oryza Sativa Japonica	Os02g0651300	*Gossypium raimondii*	Gorai.001G051800.1
	Os02g0535000		Gorai.003G111300.2
Sorghum bicolor	Sobic.004G298900	*Fragaria vesca*	mrna21066.1-v1.0-hybrid
	Sobic.004G166900.1	*Vitis vinifera*	GSVIVT01013859001
Brachypodium distachyon	Bradi3g50330.1		GSVIVT01013852001
	Bradi3g45010.1	*Trifolium pratense*	Tp57577_TGAC_v2_mRNA15638
Citrus clementina	Ciclev10032174m	*Zea mays PH207*	Zm00008a022489_T01
	Ciclev10012134m	*Mimulus guttatus*	Migut.F01209.1
Citrus sinensis	orange1.1g021509m		Migut.F00540.1
	orange1.1g021514m	*Musa acuminata*	GSMUA_Achr8T14990_001
Arabidopsis thaliana	AT5G61670.1		GSMUA_Achr11T00320_001
	AT5G06130.2	*Cucumis sativus*	Cucsa.129150.2
Solanum lycopersicum	Solyc03g093830.2.1	*Malus domestica*	MDP0000300938
	Solyc09g010110.2.1		MDP0000136536
Solanum tuberosum	PGSC0003DMT400022863		MDP0000158020
Zea mays	GRMZM2G039089_T03	*Prunus persica*	Prupe.2G270800.1
Brassica rapa	Brara.I00670.1	*Panicum hallii*	Pahal.A02767.1
Brassica oleracea var. *botrytis*	Boor	*Panicum virgatum*	Pavir.Ab00376.1
Selaginella moellendorffii	92027		Pavir.Aa01191.1
Physcomitrella patens	Pp3c19_22560V3.2	*Setaria italica*	Seita.1G255000.1
Amaranthus hypochondriacus	AHYPO_019109-RA	*Setaria viridis*	Sevir.1G259200.1
		Brachypodium stacei	Brast04G119900.1
Daucus carota	DCAR_020166		
		Oropetium thomaeum	Oropetium_20150105_02909A
	DCAR_009463		
		Kalanchoe laxiflora	Kalax.1325s0004.1
Glycine max	Glyma.12G003500.3		
		Kalanchoe fedtschenkoi	Kaladp0493s0008.1
	Glyma.09G233300.2		
		Aquilegia coerulea	Aqcoe3G260500.1
	Glyma.02G201600.1		
		Zostera marina	Zosma116g00200.1
Boechera stricta	Bostr.26833s0067.1		
		Carica papaya	evm.model.supercontig_13.235
Capsella rubella	Carubv10026827m		
		Amborella trichopoda	evm_27.model.AmTr_v1.0_scaffold00002.208
Eutrema salsugineum	Thhalv10004649m		
		Linum usitatissimum	Lus10016713
Theobroma cacao	Thecc1EG014816t1		
		Spirodela polyrhiza	Spipo5G0052000
Ricinus communis	30147.m014349		
		Cucumis melo	AIZ95444.1
Phaseolus vulgaris	Phvul.011G004200.1		
		Chlamydomonas reinhardtii	Cre06.g279500.t1.1
Eucalyptus grandis	Eucgr.A02931.1		
		Volvox carteri	Vocar.0002s0133.1
Populus trichocarpa	Potri.012G114200.1		
		Ostreococcus lucimarinus	89440
Manihot esculenta	Manes.06G130100.1		
		Coccomyxa subellipsoidea C-169	6492
	Manes.14G040800.1		
		Micromonas sp. RCC299	97450

Table S3. Primers used in this study

List of primers for sgRNA construction	
Primer name	sequences (5' to 3')
Osor target1 F	gttgCCAAATCAGCTCATTACTAA
Osor target1 R	aaacTTAGTAATGAGCTGATTTGG
Osor target2 F	gttgTTGATTCCATCAATATCCAG
Osor target2 R	aaacCTGGATATTGATGGAATCAA
Osor target3 F	gttgGAAGTATATATACTTACCAG
Osor target3 R	aaacCTGGTAAGTATATATACTTC

List of primers for HMA genotyping and RT-PCR	
Primer name	sequences (5' to 3')
Osor 3rd exon F	GGATCTCAGTTGATGTGCCTGAAGG
Osor 5th exon R	GGCAAATGGACACTGCGGATGAAG
Osor 1st exon F	GTCGTTCGGCTCTGGTGATG
Osor 8th exon R	TTAATCAAACGGATCAATTCGAGGGTC
